# Microplastic-Mediated Heavy Metal Uptake in Lettuce (*Lactuca sativa* L.): Implications for Food Safety and Agricultural Sustainability

**DOI:** 10.3390/molecules30112370

**Published:** 2025-05-29

**Authors:** Bhakti Jadhav, Agnieszka Medyńska-Juraszek

**Affiliations:** Institute of Soil Science, Plant Nutrition and Environmental Protection, Wroclaw University of Environmental and Life Sciences, 53 Grunwaldzka Str., 50-357 Wroclaw, Poland; bhakti.jadhav@upwr.edu.pl

**Keywords:** microplastics, heavy metals, *Lactuca sativa*, soil contamination, bioavailability, agricultural sustainability, metal accumulation

## Abstract

This study investigates how different types of microplastics (MPs)—fibers, glitter, plastic bags, and plastic bottles—influence heavy metal uptake in lettuce (*Lactuca sativa* L.), a commonly consumed leafy vegetable. A controlled eight-week pot experiment was conducted in a greenhouse using contaminated loamy sand soil (polluted with Cd, Pb, Cu, and other metals) collected from a smelter-impacted area. Microplastics were added at a concentration of 70–80 mg/kg, and lettuce seedlings were grown under phytotron conditions (22 ± 2 °C, 60 ± 5% RH, 16 h light/8 h dark) without fertilizers or external contaminants. Plant roots and shoots were harvested, and heavy metals were analyzed via MP-AES and ICP-MS. The results showed that MPs altered heavy metal mobility, bioavailability, and plant uptake. Copper accumulation in leaves decreased substantially across MP treatments, from 80.84 mg/kg in the control to 26.35 mg/kg (glitter), whereas lead and cadmium concentrations increased significantly in roots under fiber and glitter exposure (Pb increased from 12.13 mg/kg to 33.57 mg/kg and Cd from 1.70 mg/kg to 2.05 mg/kg in fiber treatment). Cobalt accumulation in leaves increased under the plastic bag treatment, indicating MP-specific metal interactions. Root growth was also affected, with fibers promoting elongation and plastic bottles restricting it. Sequential extraction revealed that MPs modified metal partitioning in soil, with Pb and Ni more strongly retained in stable fractions under some treatments. Observed trends in soil pH and organic matter content were associated with changes in metal mobility, highlighting the potential role of soil properties in mediating microplastic–metal interactions. These findings highlight the role of MPs as mediators of heavy metal transport in crops and underscore the need for clear regulatory guidelines that limit microplastic contamination in agricultural soils and promote routine monitoring to safeguard food safety and crop health.

## 1. Introduction

Microplastics, defined as plastic particles smaller than 5 mm, have become a widespread environmental contaminant due to the rapid increase in plastic production (over 390 million tons annually as of 2021) and ineffective waste management [1]. These particles originate from primary sources, such as industrial abrasives and microbeads in personal care products, or secondary sources resulting from the degradation of larger plastic debris [2]. Microplastics are extensively distributed in terrestrial ecosystems, particularly in agricultural soils, where they can alter soil physicochemical properties, disrupt microbial communities, and influence nutrient cycling [3]. For example, de Souza Machado et al. [4] reported a 25% decrease in soil aggregation and a 15% decline in microbial biomass in MP-contaminated soils. Recent studies indicate that microplastics in soil significantly affect plant growth, water retention capacity, and nutrient availability, which in turn can impact overall soil fertility and crop yield [5]. In polluted environments, microplastics frequently coexist with heavy metals such as cadmium, lead, and copper, which pose significant risks to soil health and crop productivity [6]. Microplastics have been shown to influence the bioavailability and mobility of heavy metals in soil, potentially enhancing their uptake by plants [7]. However, the extent and mechanisms of these interactions remain poorly understood, particularly regarding variations in microplastic types (e.g., fibers, glitter) and their impact on metal accumulation in crops [7]. Studies have demonstrated that microplastic surfaces act as sorption sites for heavy metals, affecting their desorption kinetics and accumulation patterns within plant tissues [8]. For instance, Wang et al. [9] found that cadmium adsorption increased by 30% in the presence of polyethylene microplastics. Recent research by Wang et al. [10] has shown that the presence of microplastics in soil can significantly alter the mobility and bioavailability of heavy metals, leading to increased uptake in plants. Microplastics can alter heavy metal transport through multiple pathways. Surface adsorption allows heavy metals to bind onto microplastic surfaces, influencing their mobility and bioavailability. Root penetration occurs when microplastics interact with root exudates and disrupt normal uptake processes. Additionally, microplastics may act as carriers, facilitating metal transport within plant tissues by forming metal–polymer complexes [11]. Moreover, the weathering and fragmentation of microplastics can modify their surface properties, increasing their affinity for metal binding and influencing plant exposure levels [5]. These interactions are influenced by soil characteristics such as organic matter content, moisture levels, and electrostatic charge, which determine the extent of heavy metal adsorption and desorption [8]. A recent study by Liu et al. [12] demonstrated that aged microplastics in agricultural soils can exacerbate the uptake of heavy metals by plants, highlighting the complex interactions between microplastics, soil properties, and plant health.

Lettuce (*Lactuca sativa* L.) is a widely consumed leafy vegetable valued for its nutritional content and rapid growth. Its shallow root system and sensitivity to environmental conditions make it a suitable model for studying the uptake of soil contaminants, including heavy metals and microplastics [5]. In a recent experiment, Lian et al. [13] found that polystyrene microplastics reduced lettuce root length by 18% and leaf chlorophyll content by 12%. Shi et al. [14] reported that foliar exposure to microplastics disrupts lettuce metabolism and negatively interferes with symbiotic microbial communities, potentially compromising plant health and nutritional value. Furthermore, microplastics in soil may alter metal bioavailability and uptake, yet the specific interactions between microplastics, heavy metals, and lettuce remain inadequately explored [15,16]. Furthermore, the composition of microplastics plays a critical role in their interaction with heavy metals, as different polymer types exhibit varying adsorption capacities and affinities. Fibers composed of polyester (PEs), and plastic bags, primarily made of low-density polyethylene (LDPE) or high-density polyethylene (HDPE), may act as strong adsorbents, influencing metal retention and mobility differently than glitter, which consists of polyethylene (PE), or plastic bottles, predominantly composed of polyethylene terephthalate (PET). For example, Han et al. [17] reported that LDPE enhanced Cu mobility by 40%, while PET showed no significant effect on metal sorption. These variations affect metal bioavailability and subsequent plant uptake [18]. A study by Zhang et al. [19] highlighted that different types of microplastics can significantly influence the accumulation and transport of heavy metals in plants, emphasizing the need to consider microplastic composition in environmental risk assessments. The simultaneous presence of microplastics and heavy metals in agricultural soils presents an emerging concern for crop safety and productivity. Existing studies suggest that microplastics can modify soil characteristics such as pH and cation exchange capacity, thereby influencing the mobility of heavy metals [8,16]. However, the extent to which different microplastic types impact metal accumulation in edible crops remains unclear. Given the increasing global consumption of lettuce and its crucial role in human nutrition, elucidating the impact of microplastics on heavy metal uptake in this crop is essential for food safety and environmental sustainability.

This study aims to investigate how different types of microplastics, including fibers, glitter, and plastic fragments, influence the uptake and distribution of heavy metals in lettuce. Specifically, the research seeks to evaluate the impact of microplastics on soil heavy metal bioavailability, assess differences in heavy metal accumulation between lettuce roots and leaves, and determine whether microplastics facilitate or inhibit heavy metal mobility within soil–plant systems. It is hypothesized that microplastics alter rhizosphere properties, thereby influencing the bioavailability and uptake of heavy metals in lettuce. The extent of this impact is expected to vary based on MP type, with some enhancing heavy metal mobility while others act as potential adsorbents, reducing metal uptake by plants. This study addresses critical knowledge gaps regarding the role of microplastics in heavy metal uptake by crops. By clarifying the risks posed by microplastics in agricultural soils, the findings highlight implications for food safety, the necessity of improved farming and waste management practices, and the need for policy measures to regulate plastic and metal contaminants. The study provides actionable insights to support sustainable agriculture and environmental protection efforts.

## 2. Results

### 2.1. Heavy Metal Uptake by Lettuce

This study investigates how different microplastic types (P1: fiber, P2: glitter, P3: fragmented plastic bags, P4: fragmented plastic bottles) influence heavy metal accumulation in lettuce leaves and roots, revealing that microplastics affect metal uptake to varying extents, leading to significant differences compared to the control. The significance of differences in heavy metal concentrations compared to the control is indicated by *p*-values (*p* < 0.05) in Table S1. The results reveal that microplastics have varying effects on metal uptake, with some metals showing significant increases or decreases in concentration depending on the type of microplastic present. Across all microplastic treatments, copper (Cu) levels in the leaves were significantly lower (*p* < 0.04) than in the control (80.84 mg/kg). This indicates that microplastics may interfere with the plant’s ability to absorb and transport Cu, potentially by altering soil properties or metal bioavailability. The most pronounced reductions in Cu concentration were observed under the P2 and P4 treatments, where Cu levels dropped to 26.35 mg/kg and 30.85 mg/kg, respectively. Several treatments, particularly P1, P2, and P4 (*p* < 0.05), resulted in a significant increase in Pb and Cd concentrations in the roots. This suggests that these microplastics may enhance the bioavailability or retention of these toxic metals in the root zone, potentially increasing the risk of heavy metal contamination in edible crops. The presence of P1 led to a significant increase in Pb (from 12.13 mg/kg to 33.57 mg/kg) and Cd (from 1.70 mg/kg to 2.05 mg/kg). Similarly, P2 increased Pb concentrations in roots to 20.34 mg/kg and Cd levels to 1.55 mg/kg. In the case of P3, a significant reduction in Co and Cu uptake was observed in the leaves, where Co levels increased from 2.84 mg/kg to 3.99 mg/kg while Cu levels dropped from 80.84 mg/kg to 40.77 mg/kg. Root metal concentrations remained largely unchanged, except for a slight increase in Cd (from 1.70 mg/kg to 2.10 mg/kg). For P4, Cu uptake in leaves was significantly reduced (from 80.84 mg/kg to 30.85 mg/kg), while Cd accumulation in roots increased (from 1.70 mg/kg to 2.21 mg/kg), further confirming the tendency of certain microplastics to facilitate heavy metal retention in plant roots.

These findings highlight the complex interactions between microplastics and heavy metals in soil–plant systems. The presence of microplastics alters the uptake of essential and toxic metals, with Cu being notably reduced in leaves and Pb/Cd concentrations increasing in roots. This suggests that microplastics could pose additional risks to food safety by modifying the way crops absorb contaminants from the environment.

**Figure 1:**
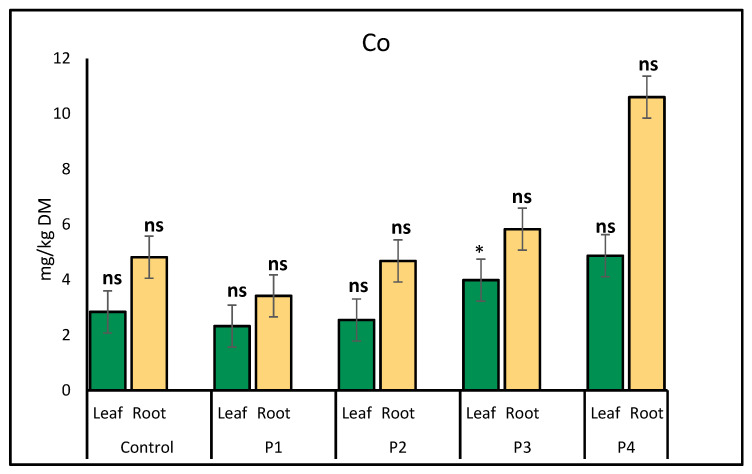
Comparison of cobalt (Co) accumulation (mg/kg) in lettuce leaves and roots under different microplastic treatments. Bars represent mean ± standard error SE (*n* = 3). Statistical comparisons with the control were made using independent *t*-tests. A significant increase in leaf Co was observed under P3 (*p* = 0.02), denoted by a single asterisk (*). All other comparisons were not statistically significant (ns, *p* > 0.05).

Cobalt accumulation in lettuce leaves and roots varied under different microplastic treatments, including P1, P2, P3, and P4, relative to the control. The results indicate differences in Co uptake between plant parts and treatments. In leaves, Co accumulation was significantly higher under the P3 treatment compared to the control, suggesting enhanced uptake. In roots, Co levels varied but did not show a clear increasing or decreasing trend across treatments (Figure 1).

**Figure 2:**
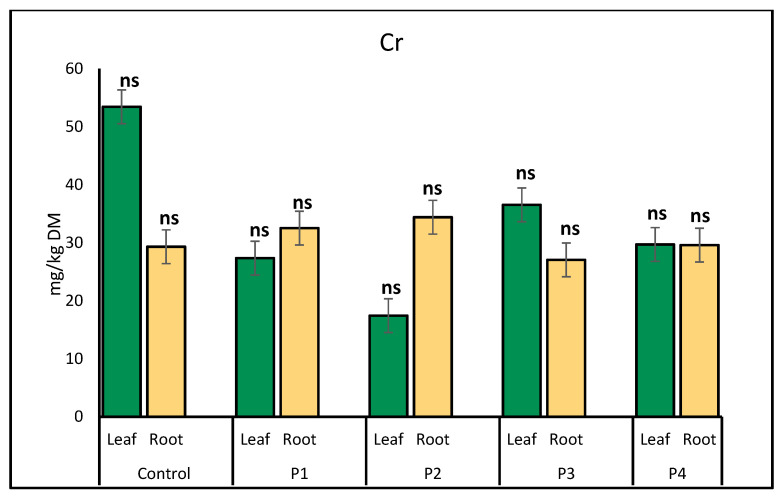
Comparison of chromium (Cr) concentrations (mg/kg) in lettuce leaves and roots across microplastic treatments. Bars represent mean ± SE (*n* = 3). No statistically significant differences were detected for either plant part (*p* > 0.05, *t*-test); all treatments are marked as non-significant (ns).

Chromium (Cr) accumulation in lettuce exhibited distinct patterns in leaves and roots under various microplastic treatments. In leaves, Cr content was highest in the control group (53.45 mg/kg) and significantly reduced across all microplastic treatments, with the lowest accumulation observed under P2 exposure (17.43 mg/kg). In contrast, Cr accumulation in roots remained relatively stable, showing slight increases under P1 (32.53 mg/kg) and glitter (34.41 mg/kg), while the P3 treatment resulted in the lowest root Cr concentration (27.05 mg/kg) (Figure 2).

**Figure 3:**
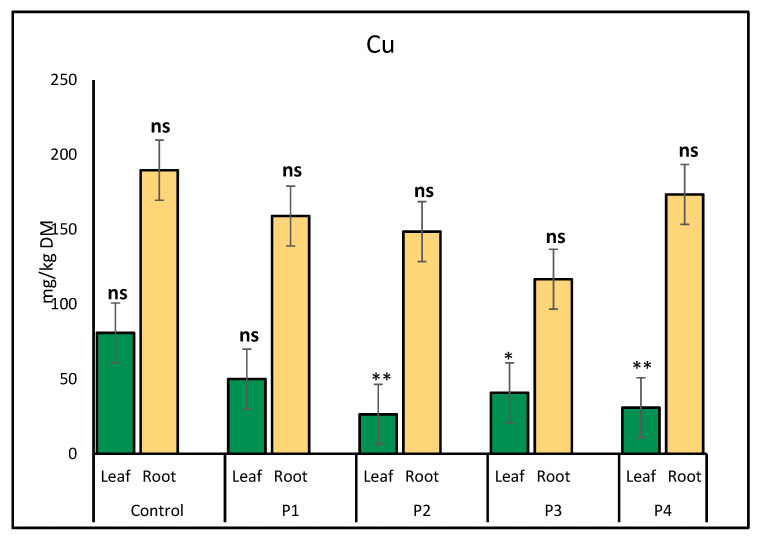
Comparison of copper (Cu) accumulation (mg/kg) in lettuce leaves and roots exposed to various microplastic types. Bars show mean ± SE (*n* = 3). Cu concentrations in leaves were significantly reduced under P2 (*p* < 0.01, **), and under P3 and P4 (*p* = 0.02 and *p* = 0.00, respectively, marked *). Root Cu values were not significantly different from the control (ns).

Copper (Cu) accumulation in lettuce was notably affected by microplastic exposure. In leaves, the highest Cu concentration was observed in the control treatment (80.84 mg/kg), while the glitter treatment led to the lowest level (26.35 mg/kg), indicating a significant decline due to microplastics. In roots, Cu accumulation increased in the control (189.69 mg/kg) and plastic bottle (173.50 mg/kg) treatments, whereas lower concentrations were observed under P1 (159.07 mg/kg) and P3 (116.79 mg/kg) treatments (Figure 3).

**Figure 4:**
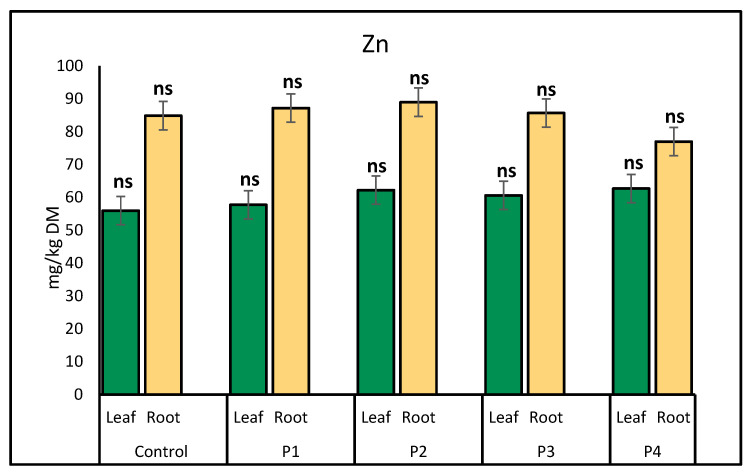
Comparison of zinc (Zn) accumulation (mg/kg) in lettuce leaves and roots in response to different microplastic treatments. Bars represent mean ± SE (*n* = 3). No significant differences in Zn concentrations were found in either plant part across treatments (*p* > 0.05, *t*-test); all values are marked as non-significant (ns).

Zinc (Zn) accumulation in lettuce showed minor variations across different microplastic treatments. In leaves, Zn levels remained relatively stable, with slight increases observed under the glitter (62.21 mg/kg) and plastic bottle (62.70 mg/kg) treatments compared to the control (55.96 mg/kg). In roots, Zn accumulation was highest under the glitter (88.98 mg/kg) and fiber (87.19 mg/kg) treatments, indicating enhanced Zn uptake under these conditions. Conversely, the lowest Zn concentration in roots was recorded under the plastic bottle treatment (76.99 mg/kg) (Figure 4).

**Figure 5:**
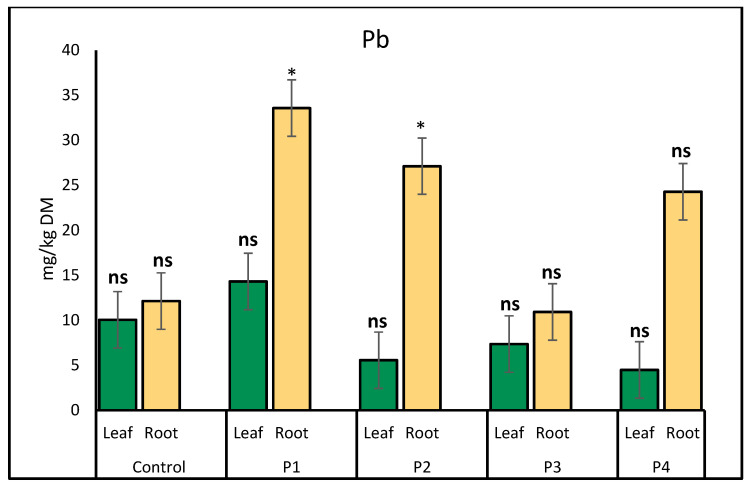
Comparison of lead (Pb) accumulation (mg/kg) in lettuce leaves and roots under different microplastic treatments. Bars represent mean ± SE (*n* = 3). Root Pb levels were significantly higher in P1 (*p* = 0.02) and P2 (*p* = 0.04), denoted by single asterisks (*). Pb concentrations in leaves and in other root treatments were not significantly different from the control (ns).

Lead (Pb) accumulation in lettuce varied notably between treatments and plant parts. In leaves, the highest Pb concentration was observed under the P1 treatment (14.31 mg/kg), while the plastic bottle treatment resulted in the lowest level (4.49 mg/kg). In roots, Pb uptake increased significantly under P1 (33.57 mg/kg) and P2 (27.12 mg/kg) treatments compared to the control (12.13 mg/kg). The P3 treatment showed the lowest Pb accumulation in roots (10.92 mg/kg) (Figure 5).

**Figure 6:**
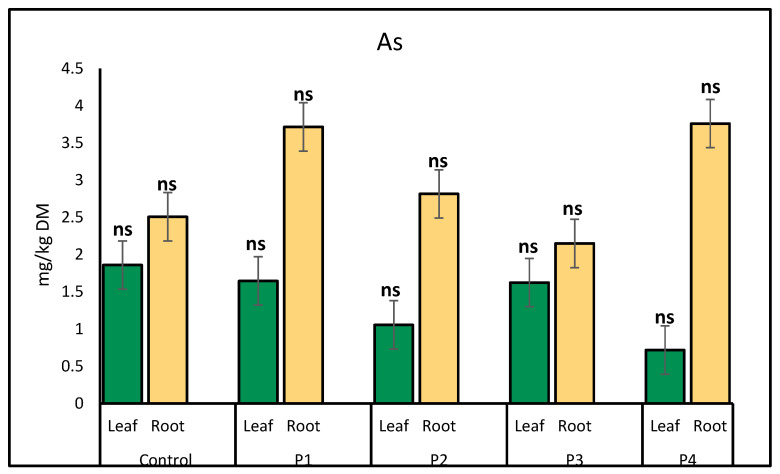
Comparison of arsenic (As) concentrations (mg/kg) in lettuce leaves and roots under various microplastic treatments. Bars represent mean ± SE (*n* = 3). No statistically significant changes in As concentrations were observed in either leaves or roots compared to the control (*p* > 0.05, *t*-test), and all treatments are denoted as non-significant (ns).

Arsenic (As) accumulation in lettuce exhibited treatment-specific variations in both leaves and roots. In leaves, the highest As concentrations were detected in the control (1.86 mg/kg) and P3 (1.62 mg/kg) treatments, while the plastic bottle treatment resulted in the lowest accumulation (0.72 mg/kg). In roots, As levels were elevated under P1 (3.72 mg/kg) and P4 (3.76 mg/kg) treatments, indicating enhanced uptake compared to the control (2.51 mg/kg) (Figure 6).

**Figure 7:**
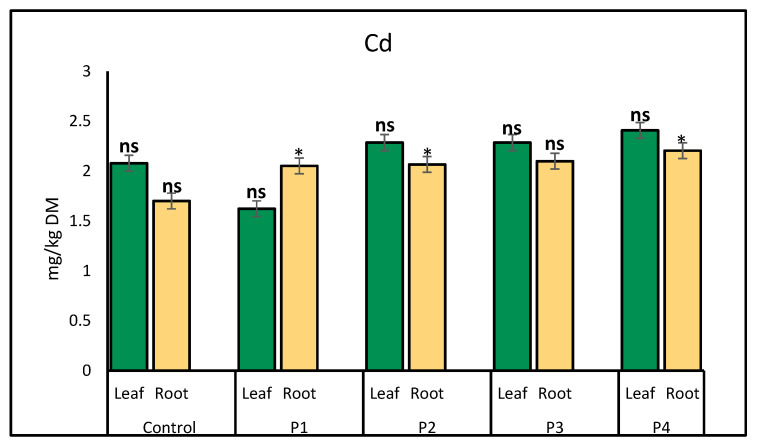
Comparison of cadmium (Cd) accumulation (mg/kg) in lettuce leaves and roots following exposure to different microplastic types. Bars represent mean ± SE (*n* = 3). Root Cd concentrations were significantly elevated under P1 (Fiber, *p* = 0.02), P2 (*p* = 0.04), and P4 (*p* = 0.05), each marked with a single asterisk (*). No significant differences were observed in leaf Cd concentrations (ns).

Cadmium (Cd) accumulation in lettuce showed relatively minor fluctuations across treatments. In leaves, Cd levels remained fairly consistent, with a slight increase observed under the plastic bottle treatment (2.41 mg/kg) compared to the control (2.08 mg/kg). In roots, the highest Cd concentration was recorded under the P4 treatment (2.21 mg/kg), while other treatments exhibited minimal variation, ranging from 1.70 mg/kg (control) to 2.10 mg/kg (P3 treatment) (Figure 7).

### 2.2. Root Growth and Biomass Responses

Root length was significantly affected by microplastic treatments when compared to the control. The control group exhibited an average root length of 9.20 ± 1.31 cm, which was not significantly different from the P1 (10.30 ± 3.41 cm) or P2 (9.62 ± 1.44 cm) treatments. However, root length under the P4 treatment (7.12 ± 1.27 cm) was significantly shorter than the control (*p* < 0.05). Among all treatments, P1 produced the longest roots (10.30 cm), while P4 resulted in the shortest (7.12 cm) (Table 1). The significantly reduced root length in the P4 treatment suggests that microplastics from plastic bottles may have a more detrimental impact on root elongation, potentially due to chemical leachates or physical interference with root growth. In contrast, P1 exhibited the longest roots, although the difference was not statistically significant when compared to the control, indicating that P1 fiber-type microplastics may not strongly inhibit root elongation and could potentially stimulate growth.

Regarding root biomass, no significant differences were observed among treatments, with values ranging from 0.34 ± 0.06 g in P4 to 0.48 ± 0.02 g in the control. This suggests that while microplastics may affect root elongation, they do not necessarily impact overall root biomass. The observed differences in root length but not in biomass may be attributed to several factors, including the physical impact of microplastics on soil structure, potential chemical leachates affecting root development, or mechanical stress imposed by rigid plastic particles. The study suggests that microplastic contamination can alter root morphology, with plastic bottle-derived microplastics showing the most negative effects, while root biomass remains largely unaffected, indicating that plants may compensate for root length changes through other physiological adaptations. Further investigation into the mechanisms behind these effects would help clarify how different microplastic types influence plant development.

**Figure 8:**
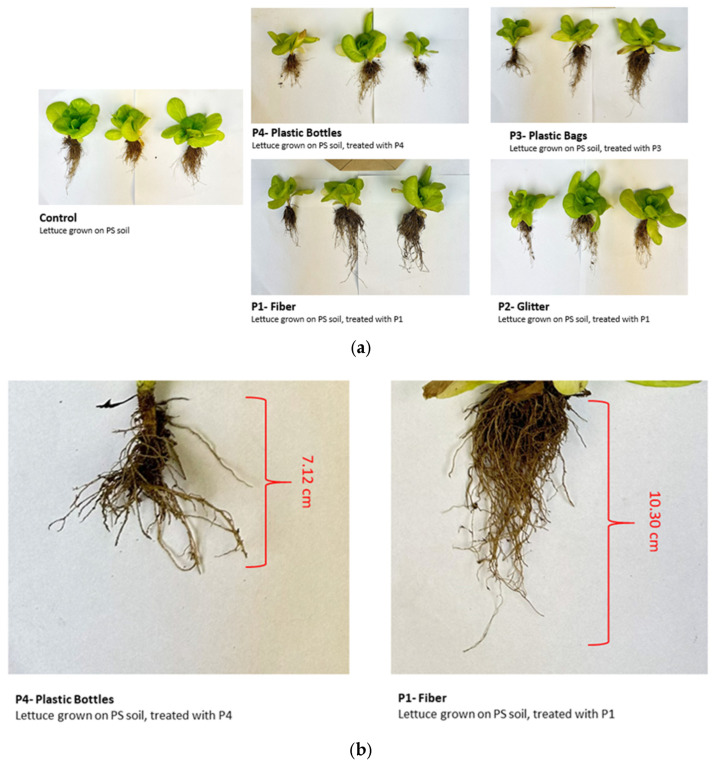
Visual observations of lettuce growth under different microplastic treatments in polluted soil (PS). The control represents lettuce grown on PS without any microplastic (MP) addition. (**a**) Lettuce grown in soil treated with plastic bottles (P4) showed the shortest root length, while (**b**) fiber (P1) resulted in the longest roots. No significant differences in root biomass were observed across treatments.

Visual observations of lettuce growth under different microplastic treatments revealed distinct morphological differences (Figure 8). In the control group, plants exhibited a moderate number of well-formed leaves with uniform green coloration and smooth surfaces, indicating healthy growth. Plants treated with P1 showed the most vigorous growth, with the highest number of leaves, broad leaf surfaces, and dark green coloration, suggesting minimal stress. In contrast, lettuce grown with P2 had fewer and smaller leaves with a slightly paler color and occasional curling, indicating mild physiological stress. The treatment with P3 resulted in visibly stressed plants, with reduced leaf number and size, pale green to yellowish coloration, and leaf surface irregularities such as curling and crinkling. The most severe effects were observed in plants exposed to P4 microplastics, which had the fewest leaves, marked reduction in leaf size, pale and sometimes distorted leaves, and signs of leaf edge damage. Lettuce grown in soil treated with plastic bottles (P4) exhibited the shortest root length (7.12 cm), indicating significant inhibition of root elongation. Similarly, plastic bags (P3) reduced root length (7.53 cm), though to a lesser extent than P4. In contrast, the fiber treatment (P1) resulted in the longest root length (10.30 cm), suggesting a less detrimental or even slightly beneficial effect on root development. The glitter treatment (P2) showed intermediate root growth, similar to the control group. Despite these variations in root length, no significant differences in root biomass were observed across treatments, indicating that while microplastics may influence root morphology, they do not necessarily impair overall biomass accumulation. These visual observations align with the quantitative data on root length and biomass, further supporting the influence of microplastic type on root development.

### 2.3. Metal Fractionation in Soil

**Figure 9:**
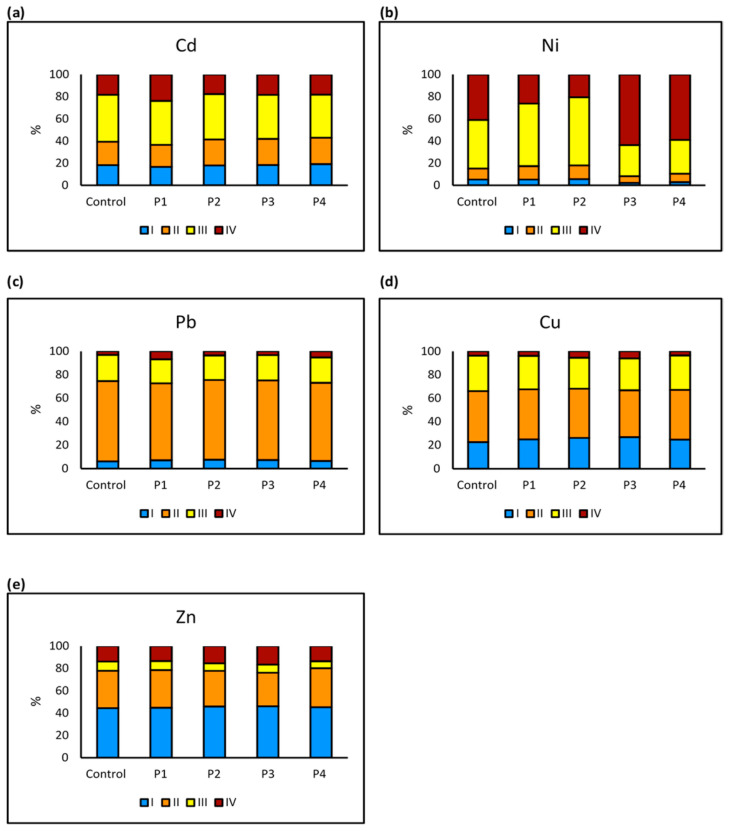
Comparison of the percentage of heavy metals (Cd, Ni, Pb, Cu, Zn) in soil sequential extraction fractions (I to IV) under different microplastic treatments: (**a**) Cd distribution, (**b**) Ni distribution, (**c**) Pb distribution, (**d**) Cu distribution, and (**e**) Zn distribution. Microplastic treatments significantly altered metal fractionation in soil, with Cd (P3, P4) and Ni (P2) affected in Fraction II, and Pb (P1–P3), Cu (P3), and Zn (P2, P3) showing reduced concentrations in Fraction III. Bars represent the percentage of total metal content (*n* = 3). Significant differences compared to the control were determined using independent *t*-tests (*p* < 0.05).

The distribution of heavy metals (Cd, Ni, Pb, Cu, and Zn) across soil fractions (I to IV) under different microplastic treatments highlights the influence of microplastics on metal mobility and stability. Cadmium (Cd) distribution reveals that Cd is primarily found in the more mobile Fractions I (exchangeable fraction and bond to carbonates) and II (reducible metals), with P3 and P4 increasing Cd availability. This indicates that P3 and P4 contribute to the enhanced mobility of Cd in soil, making it more susceptible to leaching and uptake by plants, which may pose environmental and health risks. The presence of these microplastics significantly enhances Cd concentrations in Fraction II, suggesting greater environmental risk due to increased Cd mobility. This implies that Cd becomes more bioavailable, potentially increasing toxicity in soil ecosystems and groundwater contamination (Figure 9a). Ni remains primarily in Fraction II, signifying moderate mobility, with P2 significantly reducing its retention, thereby affecting Ni stability in soil. The reduced retention of Ni under P2 treatment suggests that microplastic particles alter the soil’s ability to bind Ni, potentially increasing its release into surrounding environments (Figure 9b). Lead (Pb) shows that Pb concentrations are highest in Fractions II and III (oxidizable, bond to organic matter), suggesting moderate stability. This indicates that Pb is not highly mobile but still has a potential risk of being released from the soil, especially under external environmental influences. However, Pb retention in Fraction III is significantly reduced under P1, P2, and P3, indicating that these microplastics may contribute to Pb remobilization and potential environmental risk. The decrease in Pb retention means that lead, a toxic heavy metal, may be more readily available for plant uptake and could pose a threat to both soil quality and water systems (Figure 9c). Copper (Cu) is widely dispersed across all fractions, with the highest concentrations in Fraction III. This suggests that Cu, an essential but potentially toxic metal, has a relatively stable presence in soil, but its availability may shift under certain conditions. The presence of P3 significantly reduces Cu retention in Fraction III, implying that this microplastic type enhances Cu mobility, potentially making it more bioavailable. The increased Cu mobility due to P3 suggests that Cu could become more available for plant uptake, which may affect soil fertility and toxicity levels (Figure 9d). Zinc (Zn) is predominantly found in Fractions I and II, reflecting moderate mobility. This distribution suggests that Zn has a higher tendency to move within the soil, which could influence its bioavailability and potential for environmental contamination. Significant reductions in Fraction III Zn concentrations under P2 and P3 suggest that these microplastics influence Zn retention, potentially increasing its availability in the soil environment. This means that Zn, which is an essential micronutrient but toxic at high concentrations, may become more available for plants and microorganisms, potentially altering soil nutrient balance (Figure 9e).

### 2.4. Soil Properties and Heavy Metal Correlations

**Figure 10:**
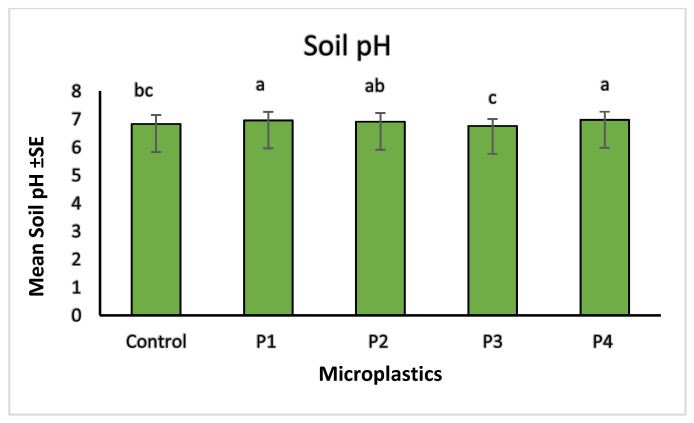
Comparison of soil pH across different microplastic treatments. Control represents the polluted soil without microplastics while P1, P2, P3, and P4 correspond to polluted soils with fiber, glitter, plastic bags, and plastic bottles, respectively. Bars represent mean values ± standard error SE (*n* = 3). A Kruskal–Wallis test (*p* = 0.051) followed by Dunn’s post hoc comparisons identified statistically significant differences among treatments. The P1 and P4 treatments sharing the same letter “a” are not significantly different (*p* < 0.05). P3 had significantly lower pH “c”. Intermediate groupings (e.g., “ab” and “bc”) indicate partial overlap without statistically significant separation.

**Figure 11:**
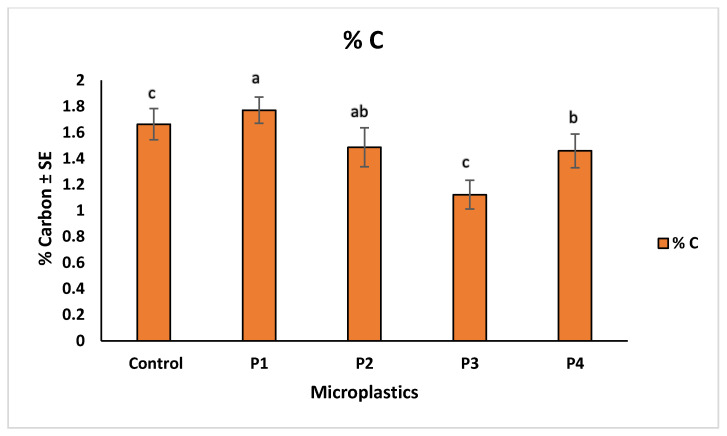
Comparison of total carbon % in soil across different microplastic treatments. Control represents the polluted soil without microplastics, while P1, P2, P3, and P4 correspond to polluted soils with fiber, glitter, plastic bags, and plastic bottles, respectively. Bars represent mean values ± standard error SE (*n* = 3). Statistical differences among treatments were assessed using the Kruskal–Wallis test (*p* = 0.039), followed by Dunn’s post hoc pairwise comparisons. Treatments assigned different letters indicate statistically significant differences (*p* < 0.05), while shared letters reflect no significant difference. P1-treated soil exhibited the highest carbon content (“a”), significantly exceeding P3 and control (“c”), while P4 (“b”) also differed from the lowest group, and P2 (“ab”) showed intermediate, non-significant variation.

**Figure 12:**
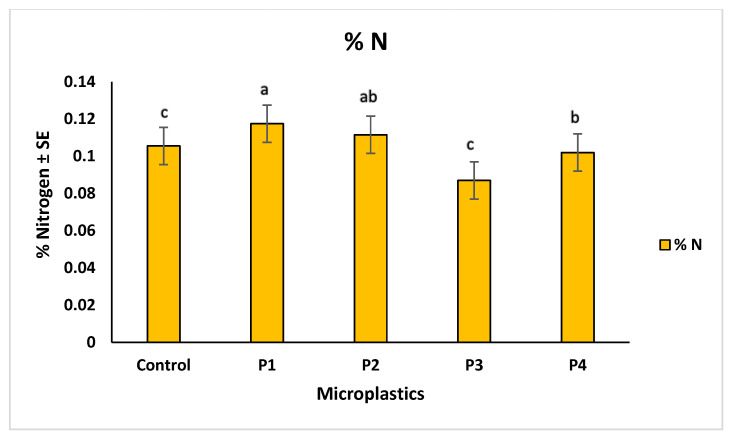
Comparison of total nitrogen % in soil across different microplastic treatments. Control represents the polluted soil without microplastics, while P1, P2, P3, and P4 correspond to polluted soils with fiber, glitter, plastic bags, and plastic bottles, respectively. Bars represent mean values ± standard error SE (*n* = 3). Statistical differences among treatments were assessed using the Kruskal–Wallis test (*p* = 0.033), followed by Dunn’s post hoc pairwise comparisons. Treatments assigned different letters indicate statistically significant differences (*p* < 0.05), while shared letters reflect no significant difference (*p* ≥ 0.05). P1-treated soil showed the highest nitrogen content (“a”), significantly higher than P3 and control (“c”), P4 (“b”) was moderately higher, and P2 (“ab”) represented an intermediate, non-significant group.

Microplastic contamination significantly affected soil chemical properties, particularly pH, total carbon (%C), and total nitrogen (%N). The control soil showed a baseline pH of 6.83. Among the treatments, soils with P3 showed the lowest pH (6.76), indicating slight acidification, while P4 resulted in the highest pH (6.98), suggesting an alkalizing effect. Soils treated with P1 and P2 had intermediate pH values of 6.96 and 6.91, respectively (Figure 10). These variations suggest that soil pH responses differed across microplastic types, possibly due to changes in aeration, or chemical interactions associated with each material. In terms of nutrient content, the highest carbon (1.77%) and nitrogen (0.11%) levels were observed in soils treated with P1, suggesting improved retention of organic matter. The lowest values were recorded for P3 treatment, with 1.12% carbon and 0.08% nitrogen, indicating possible enhancement of organic matter decomposition. P2 and P4 showed moderate values for both parameters, reflecting milder effects on soil nutrient status (Figure 11 and Figure 12). The observed changes in carbon and nitrogen content across treatments suggest that microplastics can alter nutrient cycling processes in soil, which could impact soil fertility and plant growth.

Correlation analysis between soil properties and heavy metal concentrations in soil revealed significant relationships. Soil pH showed a strong positive correlation with Cd (r = 0.887, *p* < 0.05), indicating that higher pH may increase Cd availability. Similarly, moderate positive correlations are observed between soil pH and Co, Pb, and As, indicating that Co and Pb may become more available in alkaline conditions, while the metalloid arsenic may also show increased availability. In contrast, Cu and Zn were negatively correlated with pH, implying reduced availability at higher pH levels, likely due to precipitation or reduced solubility. The correlation between pH and Cr was weak, suggesting minimal influence of pH on Cr behavior (Table 2). The carbon-to-nitrogen (C:N) ratio was also significantly associated with certain metal concentrations. A strong negative correlation with Cd suggests that increased organic matter decomposition (i.e., lower C:N ratio) may reduce Cd mobility, potentially due to binding with organic residues or microbial immobilization. In contrast, a strong positive correlation (r = 0.900, *p* < 0.05) between the C:N ratio and Cu suggests enhanced Cu availability in soils richer in organic matter, likely through complexation with organic ligands. Metals such as Co, Cr, Zn, Pb, and As showed weak correlations with the C:N ratio (Table 2). These weak correlations indicate that the availability of these metals may be affected by factors beyond organic matter decomposition. The results suggest that microplastic contamination in soils might significantly alter nutrient dynamics, with different microplastic types exerting varying influences on the soil’s chemical properties and nutrient retention.

## 3. Discussion

In this study, the different microplastic types P1, P2, P3, and P4 significantly influenced the behavior and distribution of heavy metals in the soil–plant system. Across all treatments, lettuce roots accumulated higher concentrations of metals compared to leaves, which is consistent with physiological patterns of metal uptake. As previously reported, roots often serve as the primary site of heavy metal retention, thereby limiting translocation to edible tissues [20,21].

All MP treatments significantly reduced Cu accumulation in lettuce leaves, while root Cu concentrations remained comparatively stable. This reduction in foliar Cu may be attributed to the adsorption of Cu ions onto MP surfaces or complexation with MP-associated ligands, which limits its bioavailability. These interactions likely reduce Cu’s presence in soil solution, hindering its uptake and translocation [22]. MPs can also influence rhizosphere processes such as pH, microbial activity, and organic matter dynamics, further affecting Cu mobility [23,24,25]. Significantly elevated Cd and Pb concentrations in roots were observed under the P1 and P2 treatments. Cd, known for its high mobility, appears to be further mobilized in MP-amended soils, whereas Pb, which is typically less mobile, also exhibited increased accumulation in roots, suggesting that MPs may facilitate its retention. Cd accumulation in lettuce leaves was notably reduced under the P1 treatment, whereas a slight increase was observed under P4, suggesting that fiber-based MPs may suppress Cd translocation to shoots more effectively than rigid plastic fragments. This suggests that MPs may enhance the local availability of both metals at the root–soil interface through sorption and desorption processes [26,27]. The increased availability of Cd due to MPs has also been demonstrated by Bethanis and Golia [28] and Roy et al. [29], highlighting the potential risk for edible crops grown in contaminated soils. Pb’s continued accumulation in roots confirms its limited systemic transport, yet its elevated levels under MP influence remain a noteworthy concern. Zn accumulation increased in roots under P1 and P2 treatments, while leaf Zn concentrations remained stable. This pattern suggests MPs may enhance Zn solubility or disrupt Zn sorption equilibria in the rhizosphere [4,30]. Although Zn is an essential micronutrient, its excessive accumulation may pose phytotoxic risks [31]. The weak correlation between Zn and soil C:N ratio, along with the moderate mobility observed in fractionation analysis, reflects complex MP-induced dynamics in micronutrient cycling [32,33]. In our study, the P2 microplastic treatment significantly influenced **Ni** distribution in the soil–plant system. Specifically, P2 was associated with reduced Ni retention in the soil matrix, suggesting enhanced Ni mobility and potential uptake by lettuce. This shift in soil behavior implies that MPs can alter Ni dynamics by modifying soil physicochemical properties such as pH and organic matter. These properties play a critical role in controlling metal sorption and fractionation. Our observations are consistent with those of Azeem et al. [34], who reported that combined exposure to Ni oxide nanomaterials and MPs negatively affected soybean growth and nitrogen fixation, indicating increased Ni bioavailability and toxicity under MP influence. These interactions are particularly concerning in the context of food safety, as leafy vegetables like lettuce are vulnerable to Ni accumulation and subsequent transfer through the food chain. Ammara et al. [35] highlighted similar risks, stressing the importance of understanding how MPs modulate Ni bioavailability in agricultural systems. The enhanced mobility observed in our P2 treatment underscores this concern and warrants further investigation into MP-Ni dynamics in soil–plant systems. In addition to Ni, our study also revealed treatment-specific responses in Co uptake. Notably, P3 treatment led to elevated Co concentrations in lettuce leaves, suggesting enhanced translocation from root to shoot. Although root Co concentrations did not follow a consistent trend, the increased leaf accumulation indicates that certain MP types may facilitate Co mobility and shoot translocation, likely through similar pathways affecting Ni. Given the chemical similarity between Ni and Co, it is possible that MPs influence their bioavailability via overlapping mechanisms. These findings align with Colzi et al. [36], who showed that MPs affect nutrient and metal accumulation in *Cucurbita pepo* L., and with the early observations of Moreno-Caselles et al. [37], who found that Co exposure in lettuce under soilless cultivation caused physiological stress and pollutant accumulation. Our data build upon this by showing that the presence of MPs in soil, not hydroponic systems, can similarly alter Co partitioning and increase foliar accumulation. Leaf Cr concentrations were significantly reduced across all MP treatments, especially P2, while root levels remained relatively stable. This suggests that MPs may limit Cr translocation from roots to shoots, potentially through altered Cr fractionation or interference with transport mechanisms. Slight increases in root Cr under P1 and P2 may be attributed to increased root surface adsorption or competitive uptake dynamics. Cr mobility appears to be more strongly influenced by redox conditions and ligand interactions than by pH alone [30]. While less commonly discussed in MP interactions, As showed treatment-specific accumulation patterns. Higher root concentrations were observed under P1 and P4 treatments, suggesting that certain MPs may influence As retention. MP-driven shifts in redox potential or organic matter transformation could affect As fractionation, particularly the balance between arsenite and arsenate, which impacts plant uptake [38]. Given As’s toxicity and relevance to food safety, this finding underscores the need for further study in MP-contaminated systems. In addition to metal-specific effects, MPs altered key soil and rhizosphere properties such as pH and nutrients that influence metal mobility and availability. Previous studies have shown that MPs can disturb nutrient cycling and redox balance, thereby indirectly regulating heavy metal bioavailability [39,40]. The unique surface chemistry and morphology of different MP types appear to drive distinct soil–plant responses, influencing not only metal retention and mobility but also the extent of their translocation to edible plant tissues. The cumulative findings indicate that MPs in agricultural soils can significantly modify heavy metal behavior, leading to increased root accumulation and, in certain cases, altered shoot translocation. While Cd remained mobile and readily available for uptake, the increased accumulation of otherwise less mobile metals like Pb and Co under MP influence raises concern regarding food chain transfer. Although some prior studies have explored the potential for engineered MPs to support metal immobilization, our findings emphasize the opposite, in edible crops like lettuce, MP presence can increase the risk of heavy metal accumulation. As demonstrated by Medyńska-Juraszek and Jadhav [41], this risk is particularly critical when toxic metals are retained in the root zone, potentially acting as a latent source under varying environmental conditions. Given the complexity of MP–metal–plant interactions, more research is needed to assess long-term implications for soil health and food safety in MP-contaminated environments.

Our sequential extraction analysis confirmed that microplastic (MP) types distinctly influence heavy metal fractionation and soil partitioning. Notably, P3 and P4 treatments significantly altered cadmium (Cd) distribution, shifting it toward more mobile fractions (I and II). This suggests increased Cd bioavailability and leaching potential in soils amended with these MP types. Such patterns are concerning, given Cd’s high toxicity and mobility. Our findings align with Chen et al. [42] and Yu et al. [43], who reported that MPs, particularly polyethylene-based materials, can enhance Cd bioavailability by modifying soil pH and organic matter interactions. Lead (Pb) behavior was also strongly affected by MP treatments. P1, P2, and P3 all reduced Pb retention in stable fractions (III and IV), indicating a tendency for MPs to destabilize Pb binding in soil. This remobilization effect may enhance Pb solubility and uptake potential. Our results are consistent with findings by Shirin et al. [44] and Binda et al. [45], who observed that MPs can decrease Pb stability in soils, possibly by disrupting organo-mineral associations or surface sorption equilibria. For nickel (Ni), P2 treatments led to reduced retention in Fraction II, suggesting a decline in moderately bound forms and a shift toward more exchangeable or leachable states. This observation supports our earlier findings on Ni mobility and mirrors results from An et al. [46], who demonstrated that MPs influence metal retention and release by altering ion binding dynamics in the soil matrix. In the case of copper (Cu)**,** the P4 treatment significantly increased concentrations in Fraction IV. This shift indicates complex MP–metal interactions that may either promote Cu binding in residual forms or reflect competitive displacement by other ions. Yu et al. [43] proposed that MPs can indirectly influence Cu partitioning through effects on soil cation exchange capacity and redox conditions. Zinc (Zn) partitioning was also modified by MPs, with the P2 and P3 treatments affecting Zn retention across fractions. These changes suggest increased Zn mobility, although leaf Zn concentrations remained relatively stable in our plant analysis. Nonetheless, the enhanced rhizospheric availability of Zn may still influence nutrient cycling and pose long-term phytotoxic risks if accumulation thresholds are exceeded. Collectively, these metal-specific shifts in fractionation patterns confirm that MPs depending on polymer type can significantly alter the mobility and long-term stability of heavy metals in agricultural soils. For example, while some MPs increased concentrations in stable fractions (e.g., Pb and Ni under P2 and P3), others facilitated partitioning into mobile forms (e.g., Cd under P3 and P4). These opposing trends highlight the chemical and structural diversity of MPs and their capacity to influence soil contaminant behavior through multiple pathways. Our findings provide further evidence that MPs not only impact immediate metal availability but also contribute to long-term changes in soil contaminant dynamics. This aligns with the work of Stevenson [47], who emphasized the importance of partitioning in determining metal mobility, and with Medyńska-Juraszek and Jadhav [8], who discussed the broader implications of MP-induced shifts in heavy metal bioavailability for soil health and food safety.

Our correlation analysis revealed that soil pH played an important role in regulating metal bioavailability under different microplastic (MP) treatments. A strong positive correlation was observed between pH and cadmium (Cd), indicating that higher (alkaline) pH conditions promoted Cd retention in the soil, thereby reducing its uptake by plants. In contrast, a negative correlation between pH and zinc (Zn) suggested that acidic conditions enhanced Zn mobility, increasing its potential bioavailability. Additionally, copper (Cu) showed a positive correlation with the carbon-to-nitrogen (C:N) ratio, pointing toward the role of organic matter in Cu retention, likely due to the formation of Cu–organic complexes that reduce its solubility. These findings are consistent with urRehman et al. [48], who demonstrated that alkaline soils limit Cd mobility, and Liu et al. [7], who reported that Zn availability increases in acidic soils. The observed relationship between Cu and the C:N ratio aligns with Shi et al. [49], who highlighted the role of organic matter in reducing Cu bioavailability through complexation. Beyond correlations, our results showed that microplastic presence significantly altered not only soil pH but also total carbon, and nitrogen content. Among the treatments, P3 induced soil acidification (decreased pH), while P4 led to alkalization (increased pH). P1 and P2 treatments showed intermediate pH responses. These shifts in pH likely influence nutrient availability in the soil matrix. With respect to nutrient dynamics, P1-treated soils exhibited the highest carbon and nitrogen content, suggesting potential stimulation of microbial biomass or organic matter stabilization. In contrast, P3 treatments resulted in the lowest carbon and nitrogen concentrations, which may indicate enhanced microbial decomposition or nutrient loss under acidic conditions. These changes underscore the ability of MPs to reshape soil chemical environments, with downstream effects on metal availability and plant health. Our observations are consistent with Li et al. [50], who found that polyethylene and polypropylene MPs reduced soil pH by altering microbial communities and nutrient retention. Similarly, An et al. [46] noted that MPs increased metal bioavailability, potentially enhancing microbial respiration and decomposition. These effects, taken together, suggest that microplastic-induced shifts in pH and organic matter availability can intensify metal cycling and nutrient loss, ultimately affecting soil fertility and crop productivity.

In our study, microplastic (MP) type had a notable impact on root development and soil nutrient dynamics. Plants grown in P1-treated soils exhibited the longest roots, likely due to improved soil aeration and moisture retention. In contrast, the shortest root length was observed under P4 treatment, suggesting that rigid MP fragments may physically restrict root elongation. Despite these morphological differences, root biomass did not differ significantly among treatments, indicating potential adaptive mechanisms that maintain biomass accumulation even under physical stress. These findings are consistent with observations by Mbachu et al. [51] and Li et al. [50], who reported that microplastics can alter root architecture without significantly reducing overall biomass. This suggests that plants may adjust physiologically through modified root exudation or altered microbial interactions [52,53]. However, the presence of elevated levels of toxic metals such as Co and Cu in MP-treated soils may act as additional stressors, potentially compounding the physical impact of MPs on root development [54]. P1-treated soils had the highest C and N content, likely due to minimal pH disruption. In contrast, P3 caused significant acidification and the lowest C and N levels, suggesting pH-driven processes may have influenced nutrient dynamics. This trend aligns with our findings and is supported by Li et al. [50] and Xu et al. [22], who reported that polyethylene-based MPs can lower soil pH and alter microbial activity, leading to accelerated nutrient cycling and metal solubilization. Yu et al. [43] and An et al. [46] also observed that MP-induced acidity affects microbial pathways involved in nitrogen transformation, potentially increasing nitrogen loss through leaching or denitrification under low pH conditions. These disruptions to nutrient dynamics are particularly concerning in the context of food safety. The increased accumulation of toxic metals, especially Cd and Pb in lettuce roots under MP treatments, raises the possibility of metal translocation to edible plant parts, especially in crops where roots or leaves are consumed. This reinforces concerns about the impact of MPs on soil fertility and food quality. Balkhair and Ashraf [55] emphasized the health risks posed by elevated metal concentrations in food crops, while Parolini et al. [56] highlighted the urgent need for stricter regulations on plastic waste disposal near agricultural lands. The observed differences in metal bioavailability across treatments further highlight these risks. Cd and Cr levels remained mostly unchanged across treatments, likely because they bind strongly to soil organic matter [57]. In contrast, the availability of Co, Pb, and Cu increased, especially under P3 and P4 treatments, which significantly boosted the mobility of Pb and Cu, both harmful even at low levels. This suggests that MPs may indirectly influence metal behavior by modifying soil properties such as pH and cation exchange capacity, and potentially affecting rhizosphere conditions [46,58].

These findings clearly demonstrate that MPs can modify soil physicochemical conditions in ways that enhance the mobility and bioavailability of heavy metals, particularly Cd, Pb, Co, and Cu. While morphological adaptations in roots may buffer some of the physical stress caused by MPs, the biochemical and nutritional consequences such as increased metal uptake present a far greater threat. The implications are not merely environmental but directly human; the increased bioavailability of toxic metals in soil can lead to their accumulation in food crops, elevating health risks for consumers and undermining the safety of agricultural production systems. These observations reinforce the importance of monitoring MP contamination in farmland and to better understand their compound effects with existing pollutants. While this study contributes valuable insight into MP–metal–plant interactions, further research is needed to investigate long-term effects of MP exposure under field conditions, particularly with respect to microbial mediation of metal uptake and the degradation of MPs over time. The growing presence of microplastics in agroecosystems is not just an environmental concern, it is a direct challenge to sustainable agriculture and food security. From this study, it becomes evident that microplastics, once thought to be passive residues, actively participate in reshaping the chemical and biological makeup of our soils. By modifying pH, nutrient availability and heavy metal mobility, they silently alter the health of both soil and crops. Our data highlight that attention must now shift from remediation to prevention because the cost of inaction may be measured in contaminated food, degraded soil, and compromised public health.

## 4. Materials and Methods

Polluted soil for the pot experiment was collected from an area impacted by copper smelter emissions, formerly used as arable soil and then excluded from agriculture activities due to elevated concentrations of Cd, Pb, Cu, and other metals in soil (51°42′08.0″ N 15°59′32.5″ E). To avoid additional contamination with microplastics, soil was collected from deeper layers (30–60 cm). The soil had a loamy sand texture (87% sand, 6% silt, 7% clay), with its characteristics detailed in Table 3. Microfibers were obtained during a drying process of polyester fleece blankets and sweat shirts in a tumble dryer, according to the procedure proposed by Selonen et al. [59]. Defragmented plastic bag particles were collected from decomposing material prepared using a modified version of the compostable product tests standards ASTM D6400 [60] and EN13432 [61]. Plastic bottles were cut with scissors into pieces with diameters ranging from 1.0 to 5.0 mm, according to the procedure described by Lehmann et al. [32]. Glitter beads and flakes were purchased from Sigma–Aldrich (Saint Louis, MO, USA) and commercially available sources, e.g., art shops selling glitter and plastic flakes. The size of the microplastics ranged between 100 microns and 5 mm. Lettuce (*L. sativa*) seeds were purchased commercially from the Legutko (Jutrosin, Poland) seed company.

### 4.1. Plant Material and Pot Experimental Design

**Figure 13:**
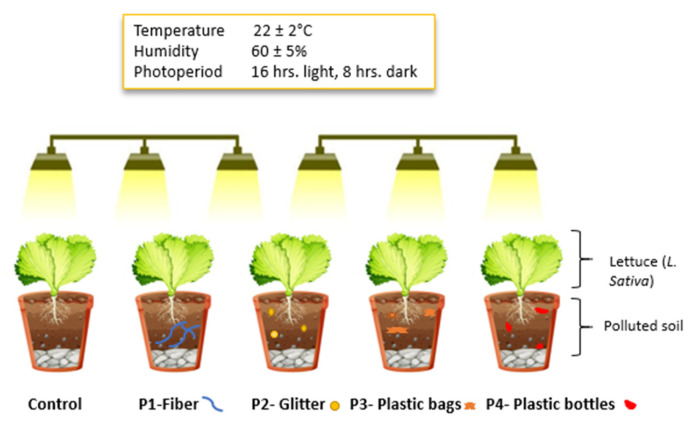
Pot experiment—lettuce (*Lactuca sativa*) growth under different microplastic treatments.

The experiment was conducted following a randomized complete block design (RCBD) with five treatment groups of pot, including a control and four MP treatments: fibers (P1), glitter (P2), fragmented plastic bags (P3), and fragmented plastic bottles (P4). Each treatment was replicated three times to ensure statistical robustness. Thus, a total of 3 pots were used per treatment, resulting in 15 pots for the entire experiment. Plants were grown under controlled greenhouse conditions with a constant temperature of 22 ± 2 °C, relative humidity of 60 ± 5%, and a photoperiod of 16 h of light and 8 h of darkness as shown in Figure 13.

### 4.2. Soil Preparation and Microplastic Treatments

The soil used in this experiment was air-dried and sieved through a 2 mm mesh to remove debris and homogenize the texture, as described by Brazauskiene et al. [62]. Prior to planting, soil physicochemical properties, including pH and carbon-to-nitrogen (C:N) ratio, were measured following the methodology outlined by Durmaz et al. [63]. The microplastics were then mixed into the 1 kg soil at a concentration of 70–80 mg to simulate realistic contamination scenarios observed in agricultural soils based on literature studies. The control group was maintained with unamended soil to serve as a baseline for comparison.

### 4.3. Plant Growth and Sampling

Lettuce seedlings were prepared before the experiment, and after two weeks from germination, they were replanted into experimental pots containing 1 kg of polluted soil. Transplanting at this stage ensured optimal root establishment and minimized early-stage stress responses. Plants were irrigated with deionized water to avoid additional metal contamination, and no fertilizers were applied to prevent interference with metal uptake. At the end of the eight-week growth period, plants were harvested, and roots and leaves were carefully separated, washed with distilled water, and oven-dried at 65 °C for 48 h prior to further analysis [64].

### 4.4. Heavy Metal Analysis

Heavy metal concentrations in plant tissues and soils were analyzed using a microwave plasma–atomic emission spectrometer (MP-AES 4200, Agilent Technologies, Santa Clara, CA, USA) and inductively coupled plasma mass spectrometry (ICP-MS), following the digestion protocol described by Gong et al. [65]. Dried leaf and root samples were ground into fine powder using a stainless-steel grinder, and approximately 0.250 g of leaves and 0.100 g of root sample were digested in a mixture of nitric acid (HNO_3_) and hydrogen peroxide (H_2_O_2_) using a microwave digestion system START-D (Milestone, Italy). Metal concentrations were quantified using external calibration curves prepared from standard solutions from Agilent Technologies (Santa Clara, CA, USA) and Merck-Sigma Aldrich (Darmstad, Germany). To ensure analysis accuracy, the certified reference material BCR 279 (produced by JRC—Joint Research Centre) was used with each sample set analyzed with the described method. All chemical analyses and biomass measurements were performed using samples collected from the three biological replicates (i.e., three pots) per treatment group, as detailed in Section 4.1.

### 4.5. Sequential Extraction of Heavy Metals in Soil

To assess the fractionation of heavy metals within soil samples, a modified sequential extraction procedure was performed based on the Bureau Communautaire de Reference (BCR) method, which categorizes heavy metals into four fractions based on their binding strength and mobility [66]. Fraction I (exchangeable fraction and bond to carbonates) was extracted using 20 mL of 0.11 mol/L acetic acid with continuous shaking for 16 h at room temperature. This fraction represents metals loosely associated with soil particles that can be readily mobilized under changing environmental conditions. Fraction II (reducible metals, bound to Fe and Mn oxide) was obtained by treating the residue from Fraction I with 20 mL of 0.1 mol/L hydroxylammonium chloride solution, followed by shaking for 16 h. This step targets metals bound to Fe-Mn oxides, which can be released under reductive conditions. Fraction III (oxidizable metals, bound to organic matter) was extracted by digesting the residue from Fraction II with 10 mL of 8.8 mol/L hydrogen peroxide at 85 °C for 2 h. After cooling, 30 mL of 1 mol/L ammonium acetate solution (pH 2.0) was added, and the suspension was shaken for 16 h. This fraction includes metals associated with organic matter and sulfides, which are mobilized under oxidizing conditions. Fraction IV (residual metals) was determined through strong acid digestion of the residue from Fraction III using aqua regia (7.0 mL hydrochloric acid and 2.3 mL nitric acid). This fraction represents metals strongly bound within the mineral matrix, which are not readily bioavailable for plant uptake. Metal fractionation was calculated and presented as a share of each fraction in the total amount of metal calculated as a sum of Fractions I, II, III, and IV.

### 4.6. Statistical Analysis

All data were analyzed using statistical software (SPSS v.26). Independent *t*-tests were performed to assess the significance of microplastic treatments on heavy metal accumulation in lettuce leaves and roots. Differences between control and microplastic treatments were evaluated at a significance level of *p* < 0.05. One-way ANOVA was used to compare the effects of different microplastic treatments on lettuce root length and biomass. Pearson correlation analysis was applied to examine the relationships between soil physicochemical properties (pH, C:N ratio) and heavy metal concentrations in plant tissues. Statistical analysis using the Kruskal–Wallis test (*p* < 0.05), a non-parametric method suitable for comparing groups with non-normally distributed data, revealed significant differences in soil pH, %C, and %N among the microplastic treatments, where Dunn’s post hoc pairwise comparisons were conducted and treatments were assigned distinct letters (a, b, c) to indicate statistically significant groupings (*p* < 0.05).

## 5. Conclusions

This study demonstrates that microplastics (MPs) can significantly alter heavy metal dynamics in the soil–plant system, with clear implications for food safety and environmental health. Among all treatments, fiber and glitter microplastics notably increased Pb and Cd accumulation in lettuce roots, while Cu uptake in leaves was consistently reduced. These shifts suggest that MPs may enhance the mobility and bioavailability of toxic metals, promoting their accumulation in root tissues and potentially limiting translocation to edible parts. Furthermore, metal partitioning in soil was strongly influenced by MP type. Glitter and plastic bag treatments led to increased stabilization of Pb and Ni, indicating a risk of long-term immobilization or remobilization of metals in contaminated soils. Such changes can affect soil fertility, nutrient cycling, and plant growth. Soil pH and organic matter content were closely linked to metal availability, highlighting how MPs may indirectly affect soil health through changes in physicochemical properties. The presence of MPs in agricultural soils, combined with their potential to act as carriers for heavy metals, raises concerns over crop contamination and human exposure. Overall, this study emphasizes the urgent need for improved plastic waste management and long-term research to assess the ecological and health impacts of microplastic pollution in agroecosystems.

## Figures and Tables

**Table 1:** Lettuce root length and root biomass across microplastic treatments.

Microplastic Treatment	Root Length (cm)	Root Biomass (gm)
Control	9.20 ± 1.31 ab	0.48 ± 0.02 a
P1 (Fiber)	10.30 ± 3.41 a	0.37 ± 0.27 a
P2 (Glitter)	9.62 ± 1.44 ab	0.43 ± 0.15 a
P3 (Plastic Bags)	7.53 ± 2.48 ab	0.39 ± 0.01 a
P4 (Plastic Bottles)	7.12 ± 1.27 b	0.34 ± 0.06 a

Mean values with the same letter (e.g., “a”) are not significantly different, while different letters (“a” vs. “b”) indicate a significant difference (*p* ≤ 0.05). “ab” means the value falls between both groups, showing no significant difference from either. Tukey’s HSD test (*p* ≤ 0.05) determined these groupings.

**Table 2:** Correlation between soil properties (pH, C:N ratios) and heavy metal data.

Soil Properties				Heavy Metal			
	Cd	Co	Cr	Cu	Zn	Pb	As
Soil pH	0.887 *	0.666	0.298	−0.095	−0.455	0.623	0.618
C:N ratio	−0.680	−0.200	−0.030	0.900 *	−0.201	0.136	0.419

Correlation coefficients between soil properties and heavy metal concentrations. An asterisk (*) indicates a statistically significant correlation (*p* < 0.05). Positive values denote direct relationships, while negative values indicate inverse relationships. The correlation coefficient (r) measures the strength and direction of associations, with values closer to ±1 representing stronger correlations.

**Table 3:** Characteristics of soil used for the experiment.

Characteristics	Values
Texture	loamy sand (87% sand, 6% silt, 7% clay).
pH	6.83
TOC	1.66%
TN	0.10%
C/N	15.8
Total Cu	354.6 mg/kg
Total Pb	147.4 mg/kg
Total Ni	18.8 mg/kg
Total Zn	68.5 mg/kg
Total Cd	13.0 mg/kg
Total Cr	30.0 mg/kg

TOC—total organic carbon; C/N—carbon-to-nitrogen ratio; TN—total nitrogen; total Cu, Pb, Ni, Zn, Co, Cd, Cr—after sample digestion in 65% nitric acid.

## Data Availability

The original contributions presented in the study are included in the article; further inquiries can be directed to the corresponding author.

## Supplementary Materials

The following supporting information can be downloaded at: https://www.mdpi.com/article/10.3390/molecules30112370/s1, Table S1: The consent of heavy metals in lettuce in the presence of different microplastics.

## Abbreviations

The following abbreviations are used in this manuscript:

MP	Microplastic
PE	Polyethylene
PEs	Polyester fibers
LDPE	Low-Density Polyethylene
HDPE	High-Density Polyethylene
PET	Polyethylene Terephthalate
*L. sativa*	*Lactuca sativa* (scientific name for lettuce)
U.S.	United States
TOC	Total Organic Carbon
C/N or C:N	Carbon-to-Nitrogen ratio
TN	Total Nitrogen
RCBD	Randomized Complete Block Design
ICP-MS	Inductively Coupled Plasma Mass Spectrometry
MP-AES	Microwave Plasma—Atomic Emission Spectrometry
HNO₃	Nitric Acid
H₂O₂	Hydrogen Peroxide
BCR	Bureau Communautaire de Référence (Community Bureau of Reference)
Fe-Mn	Iron–Manganese
SPSS	Statistical Package for the Social Sciences
ANOVA	Analysis of Variance
USA	United States of America
JRC	Joint Research Centre
P1	Fiber
P2	Glitter
P3	Plastic Bags
P4	Plastic Bottles
As	Arsenic
Cd	Cadmium
Co	Cobalt
Cr	Chromium
Cu	Copper
Ni	Nickel
Pb	Lead
Zn	Zinc
*p*-value	Probability value
%C	Percent Carbon
%N	Percent Nitrogen
pH	Potential of Hydrogen
