# Supplementary material for: Microplastic-Mediated Heavy Metal Uptake in Lettuce (Lactuca sativa L.): Implications for Food Safety and Agricultural Sustainability
Source: Molecules. 2025 May 29;30(11):2370. doi: 10.3390/molecules30112370 (PMC12156904; doi:10.3390/molecules30112370)
Supplement: Supplementary file 1 [file molecules-30-02370-s001.zip › molecules-3647191-supplementary.pdf]

Supplementary Information

Table S1. The content of heavy metals in lettuce in the presence of different microplastics.

Plant Part	Microplastic Treatment		Heavy Metal (mg/kg)						
			**Co**	**Cr**	**Cu**	**Zn**	**Pb**	**As**	**Cd**
Leaves	Control	Mean ±SE	2.84±0.29	53.45±22.49	80.84±10.87	55.96±4.29	10.05±3.47	1.86±0.68	2.08±0.18
	P1 (Fiber)		2.32±1.00	27.35±34.00	49.98±46.60	57.75±64.72	14.31±16.75	1.65±1.22	1.62±0.52
		p-value	0.35	0.15	0.04*	0.41	0.28	0.38	0.26
Roots	Control	Mean±SE	4.81±0.29	29.33±21.84	189.69±10.87	84.87±4.29	12.13±3.47	2.51±0.68	1.70±0.18
	P1 (Fiber)		3.42±1.53	32.53±19.80	159.07±143.07	87.19±85.89	33.57±26.86	3.72±3.72	2.05±0.00
		p-value	0.50	0.73	0.71	0.65	0.02*	0.54	0.02*
Leaves	Control	Mean±SE	2.84±0.29	53.45±22.49	80.84±10.87	55.96±4.29	10.05±3.47	1.86±0.68	2.08±0.18
	P2 (Glitter)		2.55±0.52	17.43±15.00	26.35±19.30	62.21±59.95	5.56±8.73	1.06±1.53	2.29±0.52
		p-value	0.26	0.08	0.00**	0.21	0.13	0.19	0.21
Roots	Control	Mean±SE	4.81±0.29	29.33±21.84	189.69±10.87	84.87±4.29	12.13±3.47	2.51±0.68	1.70±0.18
	P2 (Glitter)		3.64±1.56	25.99±34.41	111.65±148.63	66.90±88.98	20.34±27.12	2.25±2.82	1.55±0.52
		p-value	0.87	0.37	0.57	0.31	0.04*	0.82	0.04*
Leaves	Control	Mean±SE	2.84±0.29	53.45±22.49	80.84±10.87	55.96±4.29	10.05±3.47	1.86±0.68	2.08±0.18
	P3 (Plastic Bags)		3.99±0.02	36.55±25.90	40.77±24.30	60.62±58.69	7.36±2.44	1.62±2.44	2.29±0.77
		p-value	0.02*	0.25	0.02*	0.21	0.26	0.40	0.24
Roots	Control	Mean±SE	4.81±0.29	29.33±21.84	189.69±10.87	84.87±4.29	12.13±3.47	2.51±0.68	1.70±0.18
	P3 (Plastic Bags)		5.83±1.41	27.05±27.05	116.79±116.79	85.68±85.68	10.92±10.92	2.15±2.15	2.10±0.00
		p-value	0.67	0.76	0.39	0.93	0.86	0.64	0.06
Leaves	Control	Mean±SE	2.84±0.29	53.45±22.49	80.84±10.87	55.96±4.29	10.05±3.47	1.86±0.68	2.08±0.18
	P4 (Plastic Bottles)		4.87±0.90	29.70±18.00	30.85±22.00	62.70±62.22	4.49±3.65	0.72±0.90	2.41±0.60

Plant Part	Microplastic Treatment		Heavy Metal (mg/kg)						
			Co	Cr	Cu	Zn	Pb	As	Cd
Leaves	Control	Mean ±SE	2.84±0.29	53.45±22.49	80.84±10.87	55.96±4.29	10.05±3.47	1.86±0.68	2.08±0.18
	P1 (Fiber)		2.32±1.00	27.35±34.00	49.98±46.60	57.75±64.72	14.31±16.75	1.65±1.22	1.62±0.52
		p-value	0.35	0.15	0.04*	0.41	0.28	0.38	0.26
		p-value	0.13	0.18	0.00**	0.18	0.08	0.07	0.12
Roots	Control	Mean± SE	4.81±0.29	29.33±21.84	189.69±10.87	84.87±4.29	12.13±3.47	2.51±0.68	1.70±0.18
	P4 (Plastic Bottles)		10.60±2.27	29.60±29.60	173.50±173.50	76.99±76.99	24.28±8.84	3.76±3.76	2.21±0.00
		p-value	0.21	0.97	0.84	0.54	0.35	0.51	0.05*

** highly significant *significant

Table S1. Heavy metal concentrations (mg/kg, mean ± SE, n = 3) in lettuce leaves and roots grown in microplastic-contaminated soils. Treatments included Control (polluted soil without microplastics), P1 (Fiber), P2 (Glitter), P3 (Plastic Bags), and P4 (Plastic Bottles). The levels of cobalt (Co), chromium (Cr), copper (Cu), zinc (Zn), lead (Pb), arsenic (As), and cadmium (Cd) were measured. Statistical differences between treatment means were assessed using independent t-tests ($p < 0.05$). Asterisks (*) denote significant differences ($p < 0.05$); double asterisks (**) indicate highly significant differences ($p < 0.01$).
